# Characteristics of Global Health Careers among Graduates of a Global Health Equity Residency Training Program in the United States

**DOI:** 10.5334/aogh.4074

**Published:** 2023-06-21

**Authors:** Wilfredo R. Matias, C. Nicholas Cuneo, Aaron Richterman, Anne G. Beckett, Alison E. Farrar, Joseph J. Rhatigan, Daniel Palazuelos

**Affiliations:** 1 Division of Infectious Diseases, Brigham and Women’s Hospital, Harvard Medical School, Boston, MA, US; 2 Division of Infectious Diseases, Massachusetts General Hospital, Harvard Medical School, Boston, MA, US; 3 Center for Global Health, Massachusetts General Hospital, Boston, MA, US; 4 Center for Community and Global Health, Johns Hopkins University School of Medicine, Baltimore, MD, US; 5 HEAL Refugee Health and Asylum Collaborative, Baltimore, MD, USA; 6 Division of Pediatric Hospital Medicine, Johns Hopkins University School of Medicine, Baltimore, MD, US; 7 Division of Hospital Medicine, Johns Hopkins Hospital, Baltimore, MD, US; 8 Center for Public Health and Human Rights, Johns Hopkins Bloomberg School of Public Health, Baltimore, MD, US; 9 Division of Infectious Diseases, Hospital of the University of Pennsylvania, Philadelphia, PA, US; 10 Department of Internal Medicine, Boston Medical Center, Boston, MA, US; 11 Department of Pediatrics, Boston Medical Center, Boston, MA, US; 12 Harvard Medical School, Boston, MA, US; 13 Department of Physics, University of Oxford, Oxford, UK; 14 Division of Global Health Equity, Brigham and Women’s Hospital, Harvard Medical School, Boston, MA, US; 15 Partners In Health, Boston, MA, US

**Keywords:** global health equity, health education, postgraduate medical training

## Abstract

**Background:**

The number of global health (GH) physician training programs in the United States has increased in the past decade. Few studies have explored the demographics of individuals in these programs, the impact of global health training on career development, and specific factors associated with whether graduates achieve a career in global health.

**Objectives:**

We aimed to describe characteristics of program graduates and quantify which previously identified factors were associated with achieving a self-defined career in GH among a cohort of graduates from one GH post-graduate training program in a highly resourced academic medical center in the United States between 2003 and 2018.

**Methods:**

We conducted a cross-sectional survey and analyzed differences between participants who self-identified as having a career in GH compared to those who did not.

**Findings:**

Among 59 individuals invited to participate, 53 (89.9%) responded to the survey. Having a GH mentor was associated with having a career in GH (OR 10.3; p = 0.004). Those who had a GH career were more likely to have a clearly-defined career path (p = 0.03), have institutional support in their current job (p = 0.00006), be able to manage the split between their GH and non-GH work (p = 0.0001), find funding to achieve their objectives in GH (p = 0.01), invest in their personal and family life (p = 0.05), and split work abroad and domestically with few challenges (p = 0.01).

**Conclusions:**

We present sociodemographic and career characteristics for graduates from a GH training program in a highly resourced academic medical center in the United States. Mentorship, institutional support, funding, ability to balance GH with non-GH work, and time spent domestically or abroad are key factors associated with successful careers in GH. If institutional funding is allocated to strengthen these aspects of GH training, we anticipate more sustained GH career development.

## Introduction

Interest in global health opportunities during post-graduate medical training in high-income countries grew rapidly in the first two decades of the 21st century, prompting the establishment of residency and fellowship-level global health training programs at multiple United States (US) academic medical centers [1,2,3]. Despite a growing number of graduates from these programs, development of formal career pathways in global health are still in their early stages. Few analyses have explored the demographics of individuals who train in these programs, the impact of these programs on their career development, and the specific factors associated with whether graduates go on to achieve a career in global health. In addition, these few analyses have been largely descriptive, so the magnitude and significance of different factors remains unknown.

We have been engaged in training global health equity leaders and practitioners via a global health equity residency training program at a highly resourced academic medical center since 2003 and sought to share our unique experiences to inform answers to these questions. Founded in 2003 and rounding its twenty year anniversary, the Doris and Howard Hiatt Residency in Global Health Equity and Internal Medicine was established at Brigham and Women’s Hospital, an academic medical center in Boston, Massachusetts, to both address the growing need for formal training in global health equity, and to serve as an exemplar program for how to train the next generation of global health leaders [4,5]. Trainees at a minimum enter the program following completion of their medical degrees and internship year. Key pillars of the program are a unique emphasis on advancing global health equity through global health care delivery and research. Over four years of training, individuals develop the skills to independently practice internal medicine and prepare for a career in global health. The global health component of the training program includes longitudinal clinical, implementation and research projects, coupled with immersive clinical rotations with partner organizations globally and domestically. Major partner training sites include, but are not limited to, sister organizations and partners of the international non-governmental organization Partners In Health, including the Indian Health Services in Navajo Nation in the Southwestern US, Inshuti Mu Buzima in Rwanda, Zanmi Lasante in Haiti, and Compañeros En Salud in Mexico. In addition to these immersive training experiences, trainees also undertake structured global health didactics and participate in global health mentoring programs and career development sessions [5,6]. They further have the option to pursue a Master’s in Public Health at the Harvard T.H. Chan School of Public Health in Boston, Massachusetts.

We previously reported findings from a qualitative study of an early cohort of 27 graduates from the first seven years of the program [7]. Findings from this study suggested that low salaries, lack of mentorship and support, and unique life challenges were barriers to achieving global health careers. To expand our understanding of these findings, we conducted a follow-up cross-sectional survey of this cohort of graduates from our global health training program between 2003 and 2018, thereby expanding the sample size and including individuals with more advanced careers. This increased the statistical power available to further employ quantitative analyses and Likert scale questions based on themes identified in the preceding qualitative work to determine the magnitude of different factors, and to see which factors were significantly associated with careers in global health.

## Methods

### Recruitment

The focus of this study was the Doris and Howard Hiatt Residency in Internal Medicine and Global Health Equity at Brigham and Women’s Hospital in Boston, Massachusetts, US. We invited all program graduates from the cohorts entering in 2003 and graduating by 2018 using a list of active e-mail addresses for all graduates in the program.

### Survey tool

The study questionnaire was designed using REDCap (Research Electronic Data Capture) hosted at Brigham and Women’s Hospital and was e-mailed to all prospective study participants [8]. Electronic consent was obtained via the online survey. Participants were informed that they could skip questions and/or sections at their discretion, and that all information would be confidential and reported in aggregate form.

The questionnaire focused on thematic areas hypothesized to be important for global health training. These thematic areas were previously explored in a qualitative study interviewing 27 graduates from the first seven years who entered the program between 2003–2009 and graduated between 2007–2013, and explored barriers and facilitators of careers in global health [7]. For this study, we designed a study questionnaire to validate previous findings in a larger cohort, evaluate if prior findings persisted over time, and measure more granular data about themes raised in the prior study. Three authors (AF, JR, DP) formulated forced-answer questions in a new instrument to generate quantitative findings for previously reported trends and themes. The first section of the survey assessed factors related to demographics, family and relationships, student loan debt, and salary. The second section used Likert item questions on a 5-point scale (“Strongly disagree”, “Disagree”, “Neutral”, “Agree”, “Strongly agree”) to assess barriers, facilitators and perceptions related to achieving a self-defined career in global health. The survey was active from May 2019 through September 2019.

### Data analysis

We presented proportions for categorical data, means, and standard deviations (SD) for normal continuous data, and medians and interquartile ranges (IQR) for non-normal continuous data. We calculated descriptive statistics for the entire cohort. To evaluate factors associated with the outcome of having a self-defined career in global health, we performed univariable analyses using Fisher’s Exact test for proportions, given the small sample size of the study, and Wilcoxon-Rank Sum test for continuous variables. Likert items were treated as ordinal data. We used the Wilcoxon-Rank Sum test to assess the relationship between Likert item responses and the outcome of having a self-defined career in global health. Multivariable analyses were not planned *a priori* given the expected small sample size of this cohort. Statistical analyses were conducted using R version 4.0.0.

### Ethics

The study was reviewed and received Institutional Review Board exemption (Protocol #: 2019P000050) from the Partners Human Research Committee at Brigham and Women’s Hospital, Boston, Massachusetts. Written informed consent was obtained via the online survey from all participants. No minors were included in the study.

### Patient and public involvement

Study participants or the public were not involved in the design, conduct, reporting, or dissemination plans of our research.

## Results

### Demographics of the study population

Fifty-nine individuals from the inaugural global health equity class entering in 2003 through the class entering in 2014, corresponding to classes graduating between 2007–2018, were invited to participate. Fifty-three (89.9%) responded to the survey, provided consent, and enrolled in the study. Twenty-two (42.3%) respondents self-identified as female and 30 (57.7%) self-identified as male. Forty-two (80.8%) respondents were married. Thirty-four (65.3%) respondents had at least one child (Table 1).

**Table 1:** Characteristics of study participants.


CATEGORY (N = NUMBER OF RESPONDENTS IF NOT 53)	N (%)

Gender (n = 52)	

Male	30 (57.7)

Female	22 (42.3)

Current relationship status (n = 52)	

Single	5 (9.6)

Married	42 (80.8)

Other	5 (9.6)

Number of children (n = 52)	

None	18 (34.6)

One	9 (17.3)

Two	19 (36.5)

Three or more	6 (11.5)

Years since graduation	

0–5 years	26 (49.1)

>5 years	27 (50.9)

Fellowship post-graduation (n = 52)	

Yes	18 (34.6)

No	34 (65.4)

Currently in Fellowship (n = 18)*	

Yes	5 (27.8)

No	13 (72.2)

Career in global health (self-reported)	

Yes	42 (79.2)

No	11 (20.8)

Positions since graduation from GH program (n = 52) (mean (SD)	1.79 (1.40)

Are you compensated for your global health activities? (n = 52)	

All are compensated	21 (40.4)

Some are compensated	12 (23.1)

None are compensated	11 (21.2)

Does not apply	8 (15.4)

Respondents with an overall career mentor (n = 52)	

Yes	35 (67.3)

No	17 (32.7)

Respondents with a mentor for their global health work (n = 52)	

Yes	31 (59.6)

No	21 (40.4)

Who most helps you sustain your career in global health?	

Supportive partner/spouse	37 (69.8)

Your children	6 (11.3)

Your friends	16 (30.2)

Your work colleagues	31 (58.5)

Your global health mentor	22 (41.5)

Other	12 (22.6)


* Only includes individuals who responded “Yes” to pursuing a fellowship post-graduation.

### Financial health

**Figure 1:**
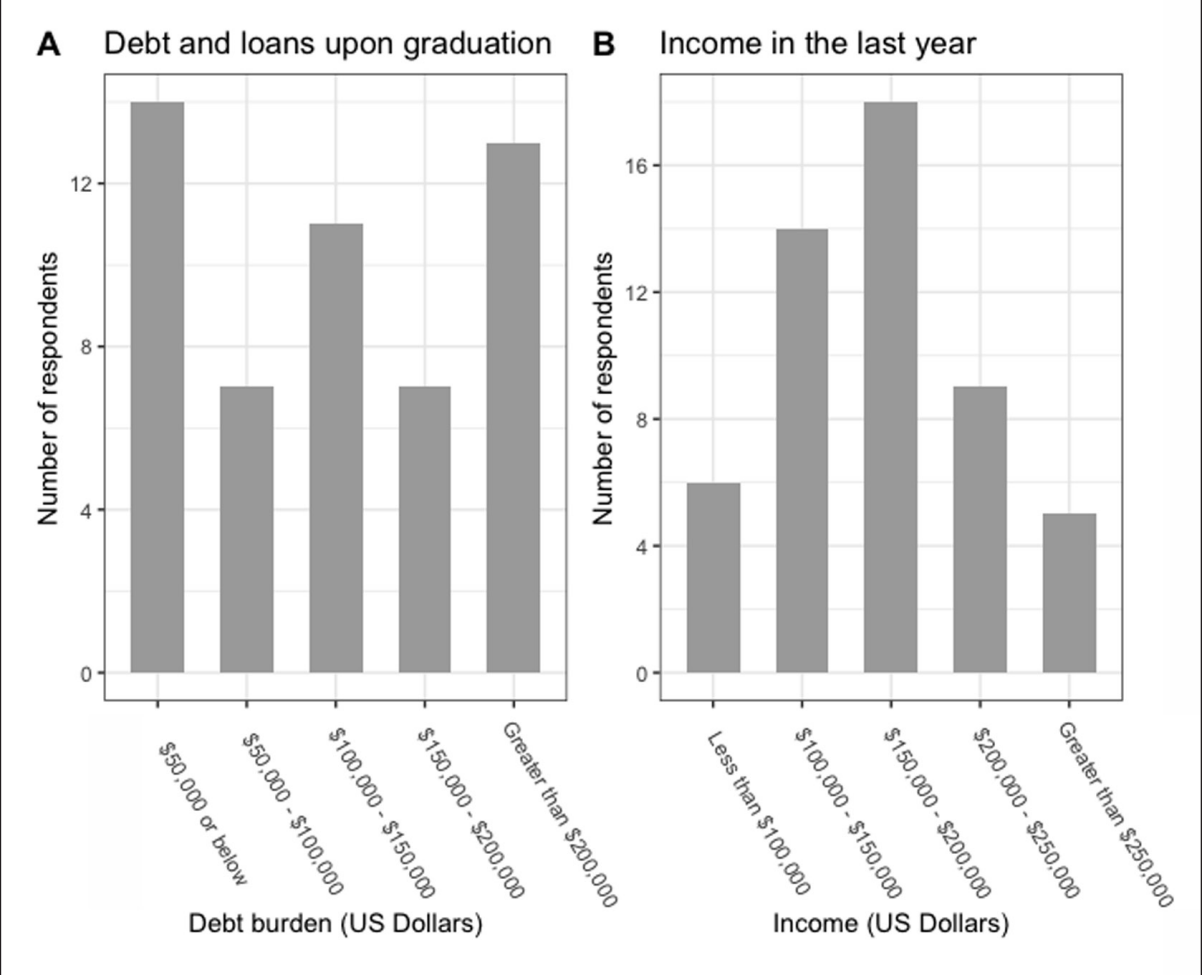
**A.** Debt and loans upon graduation of survey respondents. **B.** Income in the last calendar year of survey respondents.

The median student loan debt range at graduation was $100,000–$150,000 (Figure 1A). The median personal income range in the calendar year prior to survey response was $150,000–$200,000 (Figure 1B). The median personal income range in the calendar year prior to survey response was the same ($150,000–$200,000) when comparing individuals that were within 0–5 years post-graduation compared to those that were greater than five years post-graduation at the time of survey response. The percentage of individuals reporting a personal income greater than $150,000 in the calendar year prior to survey response was not significantly greater among those that were more than five years post-graduation at the time of survey response (69.2%) compared to those that were within 0–5 years post-graduation at the time of survey response (53.8%) (P = 0.392).

### Career trajectories and characteristics

Of all respondents, 10 (18.9%) had completed the residency training program fewer than two years ago, 16 (30.2%) between two and five years ago, and 27 (50.9%) more than five years ago. Eighteen (34.6%) went on to pursue post-graduate fellowship training. Forty-two (79.2%) respondents answered yes when asked if they considered themselves to have a career in global health (Table 1).

**Figure 2:**
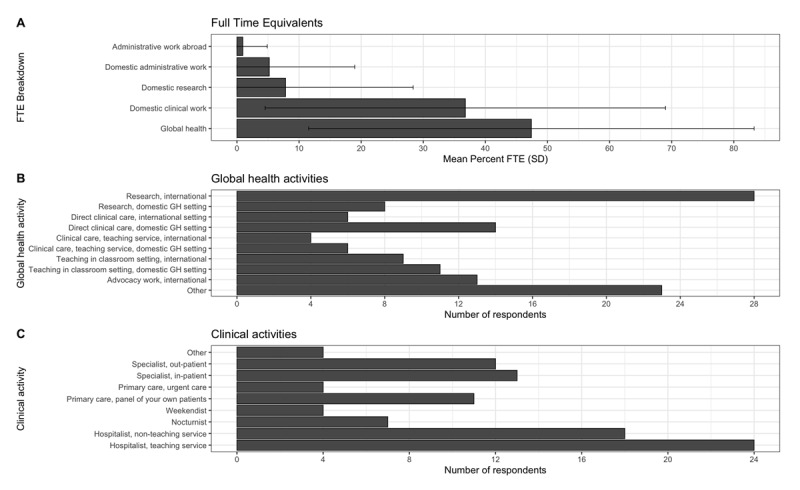
**A.** Mean Full Time Equivalent Effort of different GH activities among all participants. **B.** Number of respondents participating in different types of global health activities. **C.** Number of respondents participating in different clinical activities.

The activity to which respondents dedicated the highest mean percent full time equivalent (FTE) was global health, with a mean FTE of 47.4% (standard deviation [SD]: 35.9). This was followed by domestic clinical work with a mean FTE of 36.8% (SD: 32.2), domestic research with a mean FTE of 7.83% (SD 20.5), domestic administrative work with a mean FTE of 5.2% (SD: 13.8) and administrative work abroad with a mean FTE of 0.94% (SD: 3.93) (Figure 2A).

The most common global health work activity among respondents was international research (research conducted with partner institutions outside of the US), followed by direct clinical care in a domestic global health setting (defined as work with marginalized communities in the US, for example, working or conducting research at Indian Health Services in Navajo Nation, Southwestern US), international advocacy work, teaching in a classroom setting in a domestic setting, teaching in a classroom setting in an international setting, research in a domestic global health setting, direct clinical care internationally, and clinical care on a teaching service domestically and internationally. Twenty-three of the 43 respondents also described participating in other global health activities (Figure 2B). Clinically, most participants reported working in hospitalist roles, followed by specialist roles, and finally primary care (Figure 2C). Thirty-three (63.5%) respondents received at least partial compensation for their global health work. The mean number of positions held since graduation from the global health residency training program for all respondents was 1.79 (SD: 1.40) (Table 1).

### Mentorship

Thirty-five (67.3% of respondents) reported having an overall career mentor, compared to 31 (59.6%) who had a career mentor for their global health work (Table 1). Respondents identified several individuals who helped them sustain their career in global health. The most common individual was a supportive partner or spouse.

### Characteristics associated with a career in global health

Having a global health mentor was associated with greater odds of having a career in global health (Odds Ratio [OR] 10.3; 95% CI 1.78–112; p = 0.004). Having an overall career mentor was associated with greater odds of having a career in global health, but this difference was not statistically significant (OR 1.99; 95% CI 0.40–9.62; p = 0.47). There were no statistically significant differences between those reporting a career in global health compared to those who reported not having a career in global health in terms of time since graduation, gender, relationship status, number of children, debt, income, and pursuit of fellowship training. The mean number of positions held since graduation from the residency training program was higher among those reporting a career in global health (2.15, SD 1.33) compared to those who did not (0.46, SD 0.69) (p < 0.001) (Table 2).

**Table 2:** Characteristics associated with a career in global health.


VARIABLE	NO CAREER IN GH	CAREER IN GH	OR (95% CI)	P-VALUE

	N = 11	N = 42		

Female Gender	6 (54.5)	16 (39)	0.54 (0.11–2.52)	0.49

Current relationship status				0.57

Single	1 (9.1)	4 (9.8)		

Married	8 (72.7)	34 (82.9)		

Other	2 (18.2)	3 (7.3)		

Number of children				0.83

None	3 (27.3)	15 (36.6)		

One	2 (18.2)	3 (7.3)		

Two	4 (36.4)	15 (36.6)		

Three or more	2 (18.2)	4 (9.8)		

>5 years since graduation	6 (54.5)	21 (50.0)	0.84 (0.17–3.87)	1.00

Attended Fellowship post-graduation	5 (45.5)	13 (31.7)	0.56 (0.12–2.79)	0.48

Debt and loans > $150,000 upon graduation	5 (45.5)	15 (36.6)	0.70 (0.15–3.42)	0.73

Income > $150,000 in the last fiscal year	8 (72.7)	24 (58.5)	0.54 (0.08–2.67)	0.50

Has an overall career mentor	6 (54.5)	29 (70.7)	1.99 (0.40–9.62)	0.47

Has a GH mentor	2 (18.2)	29 (70.7)	10.3 (1.78–112)	0.004

Number of positions held since graduation (Mean [SD])	0.46 (0.69)	2.15 (1.33)		<0.001


### Differences in perceptions of global health careers

**Figure 3:**
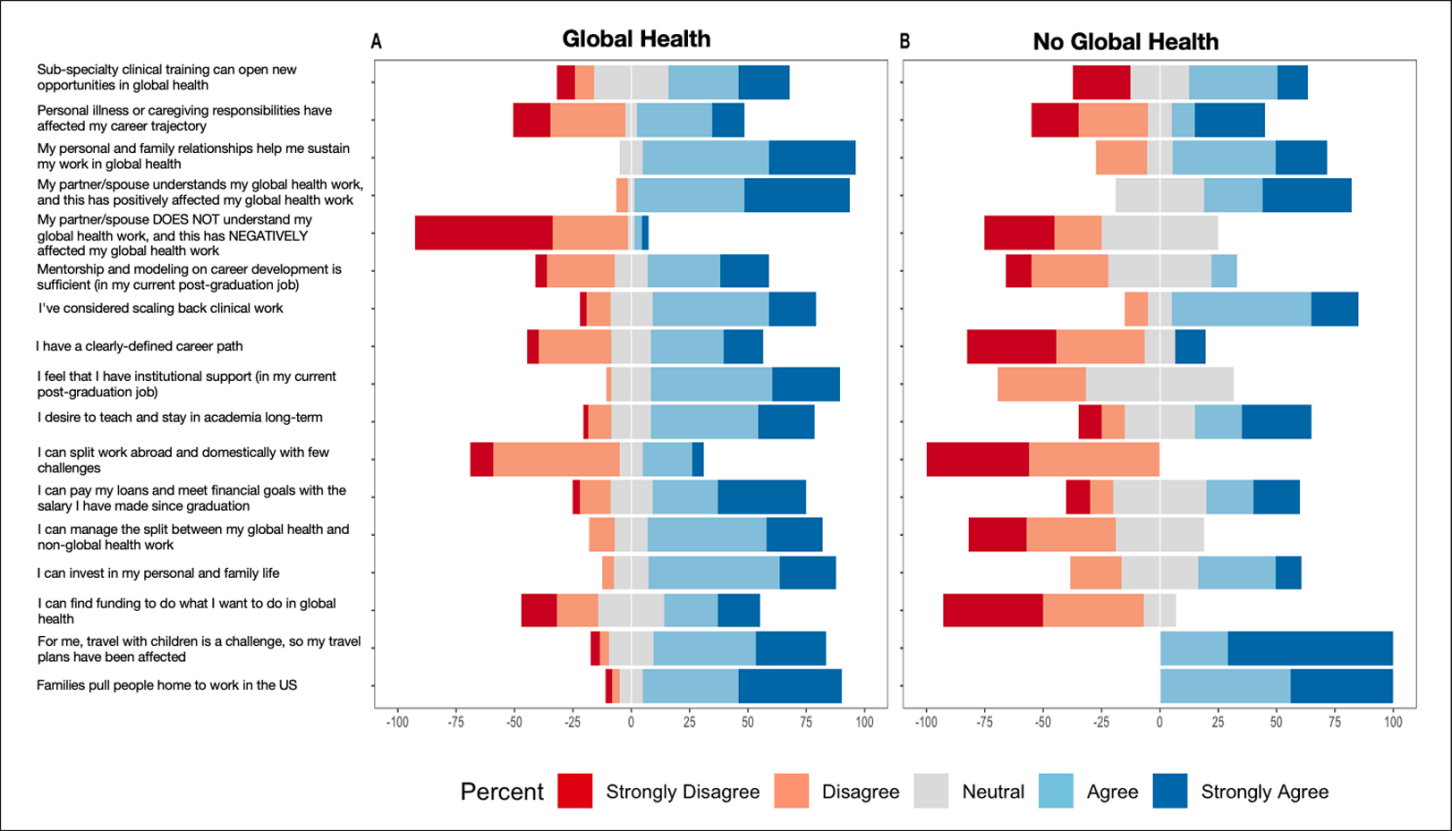
Likert item responses for individuals considering themselves to have a career in global health compared to those considering themselves to not have a career in global health.

There were key differences in perceptions regarding global health careers between individuals who reported having a career in global health compared to those who did not (Figure 3). Those who considered themselves to have a global health career were more likely to agree with the following: having a clearly-defined career path (p = 0.03), having institutional support in their current job (p = 0.00006), being able to manage the split between their global health and non-global health work (p = 0.0001), being able to find funding to do what they wanted to do in global health (p = 0.01), being able to invest in their personal and family life (p = 0.05), and being able to split work domestically and abroad with few challenges (p = 0.006).

Individuals with a global health career were less likely to be impacted by having a partner/spouse that did not understand their global health work (p = 0.03). They were also less likely to agree that travel with children was challenging and affected their plans (p = 0.04).

There were no differences in respondent perceptions regarding the sufficiency of mentorship, whether subspecialty clinical training can open new opportunities in global health, their ability to pay their loans and meet financial goals, whether their personal and family relationships helped sustain work in global health, whether one’s partner/spouse understanding of their global health work had a positive impact on their global health work, whether personal illness or caregiving responsibilities affect one’s career trajectory, and whether families pull people home to work in the US. Additionally, there were no differences with regards to participant perceptions regarding scaling back clinical work to balance career responsibilities.

## Discussion

The COVID-19 pandemic, rising global inequalities, growing calls for universal health care, and increasing globalization have put our global interconnectedness on stark display, and serve as reminders of the urgent need for health professionals who can lead in varied contexts to address problems in global health equity, both within and across borders [9]. In this study, we present further evidence on the sociodemographic factors, career characteristics, and perceptions associated with having a career in global health. These data further describe the experiences of graduates from a highly resourced global health equity medical residency training program in the US and lend greater clarity on best practices for investing in future leaders. The experiences of these graduates may serve as a bellwether for the larger enterprise of how to best develop and support leadership in global health equity.

Individuals in this cohort were diverse with regards to gender, age, relationship status, and number of children. Overall, the same percentage of respondents felt that they had a career in global health when compared to previous analyses (~80%), providing further evidence that formal global health equity training programs can achieving the goal of launching careers in global health for most graduates. Student loan debt among all graduates was high, with a median debt ranging between $100,000 to $150,00, but it was lower than the median student loan debt of $200,000 among graduating medical school students in 2020 [10]. The median yearly personal income range among graduates of the program was $150,000–$200,000. As a comparison, the mean yearly salary of hospital medicine physicians overall—the clinical focus that most global health equity residency graduates in this cohort pursue—was $276,000 in 2020 [11]; most respondents work as hospitalists because this role allows for greater flexibility to perform non-clinical work, travel, and even pro-bono work, as has been noted prior [12]. We have argued that global health practitioners based in high-income countries knowingly pay a “tax” in the form of lower salaries to participate in pro-social work, such as global health equity either domestically or globally [13]. This can also include historically under-reimbursed specialties such as pediatrics, infectious disease and primary care for underserved populations. Global health activities described in this study were heterogeneous and included clinical care, research, teaching, and administrative work domestically and abroad. The amount of time contributed to different work activities was also highly variable. These characteristics are consistent with prior findings and speak to the varying career trajectories of individuals who pursue global health training [14]. As global health programs mature and face a changing landscape, future research should focus on gathering more sociodemographic characteristics to evaluate the diverse backgrounds of students attracted to such training (e.g., race and ethnicity, country of origin, socioeconomic status) to ensure that these programs are training individuals representative of the global communities they seek to serve [15].

Among this cohort of global health equity graduates, we found that individuals who believed they had achieved a career in global health were more likely to have institutional support, to agree that they had a clearly defined career path, and to have a global health mentor. Our findings are consistent with prior literature demonstrating the importance of institutional support and mentorship in global health training [1,16]. It is likely that mentorship and institutional support play a key role in defining, clarifying, and navigating career trajectories in a field that is, despite decades of progress, still in its early stages of development and thus lacking in clearly defined metrics of success (e.g., for determining career progress and promotion) [17]. In our experience, the challenge of creating strong mentor-mentee relationships due to the limited pool of experienced global health practitioners, many of whom have limited time for mentoring activities, remains a major issue but can iteratively be alleviated if the growing alumni pool can be mobilized to give some of their time back to the program that trained them. As such, institutions should work to formalize criteria for promotion, define different models of career success in global health, and create mentorship structures that support individuals in progressing through these career trajectories. In addition to home institutional support, programs could support cross-institutional and international mentorship structures, where trainees can receive mentorship from experts in global communities away from their home institution. Examples of such cross-institutional mentorship structures in global health include the Fogarty International Center Launching Future Leaders in Global Health (LAUNCH) Research Training Program, which supports six US university consortia, each containing four US academic institutions and six or more additional collaborating institutions [18]. Calls for formalizing academic career pathways in global health at the institutional level have been growing in different fields such as oncology and emergency medicine [19,20]. Institutions must weigh their ability to formally support such pathways with formal mentorship and dedicated support when weighing the decision to establish such pathways.

Logistical challenges to a career in global health abound [13]. In our study, most individuals with a career in global health had multiple other career responsibilities, domestically and abroad. We found that individuals who had a career in global health were more likely to be able to manage the split between their global health and non-global health work, in addition to being more likely to be able to split their time abroad and domestically with few challenges. For those who work abroad, travel can become challenging, especially as families grow. Addressing these logistical challenges requires strengthening partnerships between local and global health centers to develop the necessary global supports to ensure these transitions run smoothly for such individuals. As models of global health delivery shift away from “fly-in, fly-out” to instead prioritizing local capacity strengthening and bidirectional knowledge exchange and travel, this paradigm shift may further restructure some of these traditional logistical challenges. For individuals for whom international travel is not possible, there is ample opportunity to apply a global health equity approach to domestic health challenges, as has been increasingly emphasized in the wake of the COVID-19 pandemic, which exposed critical vulnerabilities within health systems [21].

Finally, we found that individuals with a self-defined career in global health were more likely to report being able to find funding to support their career. Several studies have demonstrated the importance of funding for global health electives during training, however, knowledge regarding funding of global health careers after training is limited [1,3]. Our findings suggest that this knowledge gap can be a key barrier to a global health career. Most global health trainees will ultimately focus on direct care delivery, leadership, administration, or research. To date, funding for all these global health activities is limited, and often only available through different mechanisms that trainees may have already encountered previously to support their global health education. Individuals working in clinical, administrative or leadership roles with global organizations are generally underpaid and rely on domestic clinical work to supplement their income. Funding for global health research such as that from Fogarty International Center, the CDC, or the Bill & Melinda Gates Foundation is growing but remains limited, dynamic, and highly competitive. More funding, both at the institutional and national level, through sustainable funding mechanisms should be allocated to support global health careers. Increased stable funding would have several important consequences. First, it would allow individuals to develop the unique skillsets required to be competitive applicants for global health jobs. A recent review of global health positions demonstrated that most hiring programs identified prior overseas experience, a master’s degree, and language skills as desirable qualifications, in addition to public health and administrative skills [22]. Second, increased funding could create avenues of meaningful work that could span entire career trajectories and directly impact global health equity. Finally, and perhaps most importantly, increased funding could support the development of more equitable global health partnerships and training platforms that are more accessible to candidates who are exceptional but may not have had the same privileges as other candidates. This ability to attract an economically, racially, and nationally diverse labor pool is thus likely to result in more impactful programs. There are several successful examples of these, such as the University of Global Health Equity in Rwanda, which grounds global health equity within the local communities it serves, and the Fogarty program, which has trained thousands of health investigators from low- and middle-income countries [23,24].

This study has several limitations. First, and most importantly, this is a single-site and cross-sectional study representative of a unique global health equity medical residency training program in a highly resourced academic medical center in the US. Between different medical specialties, such as medicine versus surgery, there can be tremendous variability in the approaches taken to both global health training and developing careers. This study does not include perspectives from low- and middle-income settings. As such, findings from this program may not be generalizable to other global health training programs within the US or globally. We see value, however, in reporting here the results from this unique cohort of trainees because these trainees’ experiences may represent what is possible when trainees from a highly resourced academic US medical center choose to pursue a career in global health. Second, this study is limited by a small sample size, which precluded multivariable analyses that would allow us to hone in with greater specificity on driving factors. Finally, much has changed between the completion of this study and the time of publication. Given that the COVID-19 pandemic has significantly impacted global health practice and illuminated the severity of health inequities locally, it is possible that the demographics of individuals pursuing careers in global health, in addition to models of careers in global health, will change in the years to come. Larger, multi-institutional studies are necessary to understand global health careers more thoroughly in diverse settings and specialties.

Despite its continued growth, the field of global health education in high-income countries, and our understanding of how to prepare individuals for careers in this field, remains in its early phases. Our findings paint a picture of developing career trajectories that are limited by lack of mentorship, funding, and institutional support, which are associated with career paths that are not clearly defined and challenging for individuals to navigate successfully. Varied sources of funding can be allocated to strengthen these gaps, with potential areas of focus including the development of formal mentoring programs with individuals with established careers in global health, support for travel, and funding to support individuals throughout life changes that may affect these career transitions.

## Conclusion

In conclusion, we present sociodemographic and career characteristics of a global health equity training program cohort. Mentorship, institutional support, funding, an ability to balance global health and non-global health work, and balancing time spent domestically and abroad are key factors associated with thriving careers in global health. Recent global events have highlighted the importance of global health careers focused on health equity. Investment to support these career paths is urgently needed.

## Data Accessibility Statement

Data are available upon reasonable request from the author.
